# EsxA membrane-permeabilizing activity plays a key role in mycobacterial cytosolic translocation and virulence: effects of single-residue mutations at glutamine 5

**DOI:** 10.1038/srep32618

**Published:** 2016-09-07

**Authors:** Qi Zhang, Decheng Wang, Guozhong Jiang, Wei Liu, Qing Deng, Xiujun Li, Wei Qian, Hugues Ouellet, Jianjun Sun

**Affiliations:** 1 Department of Biological Sciences, Border Biomedical Research Center, University of Texas at El Paso, El Paso, TX 79968, USA; 2 School of Pharmaceutical Sciences, Zhengzhou University, Zhengzhou, 45001, China; 3 Department of Biological Sciences, Purdue University, West Lafayette, IN 47907, USA; 4 Medical College of China Three Gorges University, Yichang, 443002, China; 5 Department of Chemistry, University of Texas at El Paso, El Paso, TX, 79968, USA; 6 Sino-Duth Biomedical Information Engineering School of Northeastern University, Shenyang, China

## Abstract

EsxA is required for virulence of *Mycobacterium tuberculosis (Mtb*) and plays an essential role in phagosome rupture and translocation to the cytosol of macrophages. Recent biochemical studies have demonstrated that EsxA is a membrane-permeabilizing protein. However, evidence that link EsxA membrane-permeabilizing activity to *Mtb* cytosolic translocation and virulence is lacking. Here we found that mutations at glutamine 5 (Q5) could up or down regulate EsxA membrane-permeabilizing activity. The mutation Q5K significantly diminished the membrane-permeabilizing activity, while Q5V enhanced the activity. By taking advantage of the single-residue mutations, we tested the effects of EsxA membrane-permeabilizing activity on mycobacterial virulence and cytosolic translocation using the *esxA/esxB* knockout strains of *Mycobacterium marinum (Mm*) and *Mtb*. Compared to wild type (WT), the Q5K mutant exhibited significantly attenuated virulence, evidenced by intracellular survival and cytotoxicity in mouse macrophages as well as infection of zebra fish embryos. The attenuated virulence of the Q5K mutant was correlated to the impaired cytosolic translocation. On the contrary, the Q5V mutant had a significantly increased cytosolic translocation and showed an overall increased virulence. This study provides convincing evidence that EsxA contributes to mycobacterial virulence with its membrane-permeabilizing activity that is required for cytosolic translocation.

*Mycobacterium tuberculosis (Mtb*), the causative agent of tuberculosis, is considered one of the most successful pathogens. It is estimated that *Mtb* infects one-third of the world’s population and kills more than one million people each year^1^,^2^. At the early stage of infection, *Mtb* is internalized into the phagosome of host alveolar macrophage. Instead of being destroyed in the phagolysosomal compartment, *Mtb* inhibits phagosome-lysosome fusion, persists and replicates within the phagosome^3^,^4^,^5^. Recent studies have also shown that upon lysosome-phagosome fusion, *Mtb* progressively translocates from phago-lysosomes into the cytosol^6^, where *Mtb* replicates and spread to new cells^7^,^8^. Phagosomal maturation arrest and cytosolic translocation have been attributed, at least in part, to a Type VII secretion system, termed ESX-1^6^,^7^,^8^,^9^,^10^,^11^. The *esx-1* locus was first revealed by the comparative genomic studies, in which Region of Difference 1 (part of the *esx-1* locus) was found to be present in *Mtb*, but not in the attenuated vaccine strain *Mycobacterium bovis* Bacille Calmette-Guérin (BCG)^12^,^13^. Deletion of *esx-1* from *Mtb* resulted in attenuation of virulence and retention of mycobacteria in the phagosome, while transfer of *esx-1* into BCG partially restored virulence^14^,^15^,^16^,^17^,^18^,^19^,^20^,^21^. The *esx-1* locus comprises nine genes that encode the ESX-1 secretion system and two secreted effector proteins: 6-kDa early-secreted antigenic target (ESAT-6, EsxA or Rv3875) and 10-kDa culture filtrate protein (CFP-10, EsxB or Rv3874)^22^,^23^,^24^. The two proteins form a heterodimer and are secreted out of *Mtb* in a co-dependent manner^25^,^26^.

EsxA was first identified as a potent T-cell antigen in the short-term culture filtrate of *Mtb*^27^,^28^. Since then EsxA has been intensively characterized as a potential candidate of novel vaccines and immune-therapeutics against tuberculosis^29^. In recent years, EsxA has been recognized as a virulence factor essential for *Mtb* pathogenesis. It was found that *Mtb* and *M. leprae* were able to translocate from the phagolysosomal compartment into the cytosol of myeloid cells, and the cytosolic translocation was dependent on secretion of EsxA and EsxB^6^. Moreover, the ESX-1/EsxA-mediated cytosolic translocation controlled virulence of mycobacteria^7^. Using a β-lactamase-based FRET microscopy, a recent study showed that *M. marinum (Mm*) wild type strain induced phagosomal rupture and translocated to the cytosol, while the EsxA secretion–deficient strain did not^8^. Similarly, *Mtb* translocated from the phagosome to the cytosol, while BCG did not. Introduction of RD1 into BCG conferred it the ability to translocate to the cytosol. While deletion of the C-terminus of EsxA did not affect EsxA secretion, it disabled the cytosolic translocation^6^, which is consistent to the recent biochemical study that deletion of C-terminal flexible arm of EsxA caused defects in membrane permeabilization^30^. Mycobacterial cytosolic translocation was also observed in the infected mice by a FRET-based flow cytometry^11^. All of the compelling evidence suggests that EsxA plays an essential role in cytosolic translocation, and the ability of cytosolic translocation links to mycobacterial virulence.

However, the molecular details how EsxA functions in phagosome rupture and mycobacterial cytosolic translocation is not known. Several independent studies have suggested that EsxA has a membrane-lytic activity. In an earlier planar lipid bilayer study, either EsxA alone or in combination with EsxB caused disruption of the artificial membranes^17^. EsxA, but not EsxB, exhibited strong association with the liposomes and lysed the liposomes at acidic pH^31^. *Mm* infection or incubation with the purified EsxA induced pore formation on the red blood cell membranes with an estimated pore size ~4.5 nm in diameter^32^.

In the past several years, we have been systematically characterizing the membrane-permeabilizing activity of EsxA. We have found that EsxA, but not EsxB, induced leakage of liposomes in an acidic pH-dependent manner, which was accompanied by significant conformational changes and increased solvent-exposed hydrophobicity^33^. We have presented the first direct evidence that EsxA inserts into membranes with the two α-helixes forming a membrane-spanning domain^30^. More interestingly, compared to EsxA from *Mtb*, the orthologous EsxA from non-pathogenic *Mycobacterium smegmatis (Ms*) was inactive in membrane interaction, despite that they share over 76% homology^33^. This finding has raised a notion that the membrane-permeabilizing activity of EsxA is a major determinant for virulence phenotype of the *Mycobacterium tuberculosis* complex. Here, we present evidence that single-residue mutations at Q5 of EsxA either up or down regulate the membrane-permeabilizing activity, which consequently enhances or attenuates mycobacterial virulence in macrophages and in zebra fish embryos through up or down regulation of mycobacterial cytosolic translocation.

## Results

### Mutations at Q5 up or down regulated the membrane-permeabilizing activity of *Mtb*EsxA

**Figure 1:**
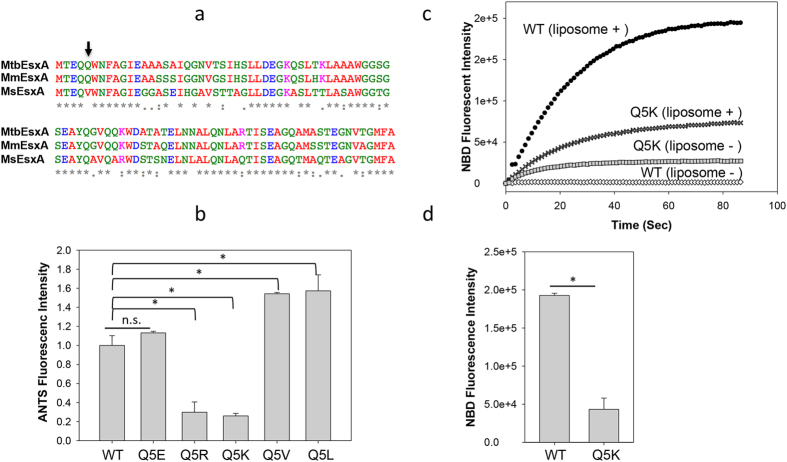
Mutations at Q5 up or down regulated the membrane-permeabilizing activity of *Mtb*EsxA. (**a**) The sequence alignment of *Mtb*EsxA, *Mm*EsxA and *Ms*EsxA. The residues are colored according to chemical nature: Red (hydrophobic), Green (polar), Blue (acidic) and Magenta (basic). Arrow: the Q5 position. (**b**) 6 μM of the indicated proteins were injected into the liposome solutions at pH 4.0. The relative membrane-permeabilizing activity was measured as ANTS fluorescence dequenching at 60 s of post-injection and quantified from at least three independent experiments. The data were presented as mean ± S.E. (n = 5, *p < 0.05). (**c**) The NBD-labeled EsxA(WT) or EsxA(Q5K) protein was incubated with or without liposomes in 20 mM TrisHCl, 100 mM NaCl (pH 7) for 30 min. Subsequently, the solution was rapidly acidified by adding 0.1 volume of 1 M NaAc (pH 4). The solution was continuously stirred in the cuvette. NBD fluorescence emission was recorded as a function of time. The representative curves from at least three repeats were shown. (**d**) The NBD emission intensity at 90 s post-acidification was calculated from three independent experiments. The background signals (liposome -) were subtracted from the signals with liposome (liposome+). The data is presented as mean ± S.E. (n = 3, *p < 0.05).

Sequence alignment shows that *Mtb*EsxA shares 93% homology with *Mm*EsxA and 76% homology with *Ms*EsxA (Fig. 1a). It promoted us to characterize the differential membrane-permeabilizing activities among different EsxA proteins. Presumably, the residues that are common or similar between *Mtb*EsxA and *Mm*EsxA, but different in *Ms*EsxA, are likely associated to membrane-permeabilizing activity. In a systematic mutagenesis analysis, we serendipitously found that mutations at Q5 were able to up- or down-regulate its membrane-permeabilizing activity using the ANTS/DPX (fluorophore and quencher) liposome leakage assay (Fig. 1b), in which the liposomes are pre-loaded with ANTS/DPX and the ANTS fluorescence is de-quenched when EsxA forms pores on the liposomal membrane to release DPX^30^,^33^. The *Mtb*EsxA(Q5V) mutant had a stronger activity than wild type (WT). Similarly, the Q5L mutant had a similar activity as Q5V, suggesting that a hydrophobic residue at this position enhances the activity. Interestingly, replacing Q5 with the basic residues K or R significantly reduced the activity, while replacing Q5 with the acidic residue E had little effect. This result was probably due to different charge states of basic and acidic residues at low pH (pH ~4), where K and R are positively charged, while E (pKa ~4.1) is (at least partly) protonated and neutral. Together, the result indicates that residues of different properties (charged, neutral or hydrophobic) at the fifth position are able to up or down regulate the membrane-permeabilizing activity of *Mtb*EsxA.

To further confirm the result, we set out to test the effects of Q5K and Q5V mutations on membrane insertion. NBD (*N*,*N*′-Dimethyl-*N*-(Iodoacetyl)-*N*′-(7-Nitrobenz-2-Oxa-1,3-Diazol-4-yl)Ethylenediamine), an environment-sensitive dye, emits fluorescence when it is shifted from an aqueous solution to a hydrophobic environment (e.g. lipid membranes). Thus, NBD fluorescence is an excellent marker for protein membrane insertion. In our previous study, we generated dozens of single-Cysteine mutants of EsxA and labeled them with NBD at the engineered Cys residues by maleimide reaction. Using NBD fluorescence, we have mapped the trans-membrane domain of EsxA, showing that both Helix 1 and 2 insert into the membrane upon acidification^30^. We have found that the NBD labeled at S35C emitted a strong fluorescence, suggesting that S35C is a good position for NBD labeling and for testing membrane insertion^30^. Thus, we introduced Q5K and Q5V mutations into the EsxA(S35C) construct. Unfortunately, EsxA(Q5V/S35C) was not expressed for unknown reasons. So we compared the NBD fluorescence (membrane insertion) between WT and Q5K. Consistent with previous result, the NBD-labeled EsxA(S35C) emitted a strong fluorescence in the presence of liposomes, indicating that it inserts into the membrane upon acidification (Fig. 1c). In contrast, the NBD-labeled EsxA(Q5K/S35C) had a significantly lower fluorescence, suggesting that it is defected in membrane insertion. Interestingly, however, we noticed that in the absence of liposomes, the NBD-labeled EsxA(Q5K/S35C) had a higher background than EsxA(S35C). This suggests that upon acidification EsxA(Q5K/S35C) undergoes a different conformational change than EsxA(S35C), in which NBD inserts into hydrophobic pockets of the protein. After calibrating off the backgrounds, EsxA(Q5K) has ~25% of membrane insertion of EsxA(WT) (Fig. 1d), which is consistent with the membrane leakage assay.

In the rest of the study, we used Q5K and Q5V to test the effects of EsxA membrane-permeabilizing activity on mycobacterial virulence and cytosolic translocation. We hypothesize that Q5K will reduces, but Q5V will enhance mycobacterial cytosolic translocation and the virulence.

### Construction and complementation of *Mm*∆EsxA/B

**Figure 2:**
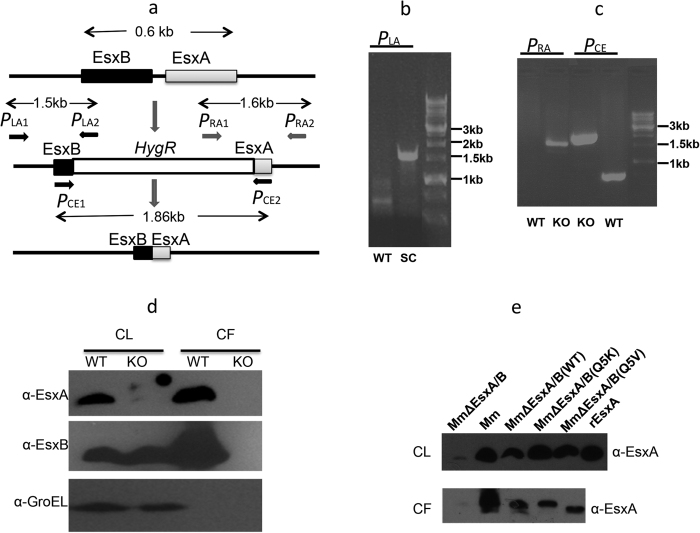
Generation and complementation of *Mm*ΔEsxA/B. (**a**) The schematic drawing of the experimental design to delete *esxB*/*esxA* by homologous recombination. The genes of *esxB*/*esxA* were replaced with a hygromycin-resistant gene cassette by allelic exchange. The hygromycin-resistant gene cassette was further removed by Cre recombinase. The primers for amplification of left arm (P_LA_1 and 2), right arm (P_RA_1 and 2) and the genes of *esxB/esxA* (P_CE_1 and 2) were labeled. (**b**) The single-crossover of left arm was confirmed by PCR using P_LA_ primers. WT: wild type; SC: single-crossover. (**c**) The knockout of *esxB/esxA* was confirmed by PCR using P_RA_ and P_CE_ primers. KO: knockout. (**d**) The total cell lysate (CL) and culture filtrate (CF) from WT and KO strains of *Mm* were loaded to SDS-PAGE and subjected to Western blot using anti-EsxA and anti-EsxB antibodies. GroEL was tested as a control to distinguish CL and CF. (**e**) Complementation of the *Mm*ΔEsxA/B strain with *esxB or esxA* genes carrying WT and Q5 mutants. EsxA proteins in CL and CF were detected by western blot using anti-EsxA antibody. A recombinant EsxA purified from *E. coli* was used as a positive control.

*Mm* causes tuberculosis-like diseases in fish and it is considered as a surrogate model for *Mtb*^34^,^35^. More importantly, *Mm* and *Mtb* share high homology in the ESX-1/EsxA system (Fig. 1a)^36^,^37^. To test the effects of Q5K on mycobacterial virulence, we generated an *esxA/esxB* knockout strain of *Mm* (termed *Mm*∆EsxA/B) by homologous recombination (Fig. 2a). Deletion of *esxA* and *esxB* was confirmed by PCR using either left-arm primers (Fig. 2b), right-arm primers (Fig. 2c), or the primers flanked the two ends of *esxA/esxB* locus (Fig. 2c). The deletion was further confirmed by probing EsxA and EsxB proteins in both cell lysate (CL) and culture filtrate (CF) in western blots (Fig. 2d). As expected, *Mm*EsxA was detected in both CL and CF of *Mm*, but not in *Mm*∆EsxA/B. Interestingly, a protein band similar to EsxB was detected in CL of *Mm*∆EsxA/B, but not in CF, suggesting that this band may be an EsxB homologous protein in CL that cross-reacted with the EsxB antibody. The *Mm*∆EsxA/B was then complemented with the plasmid pMH406 that constitutively expresses *Mtb*EsxB as well as *Mtb*EsxA WT, Q5K or Q5V respectively. Expression and secretion of the complemented *Mtb*EsxA proteins were confirmed by western blots (Fig. 2e).

### Effects of Q5K and Q5V on mycobacterial *in vitro* growth, adherence, uptake and intracellular survival in macrophages

**Figure 3:**
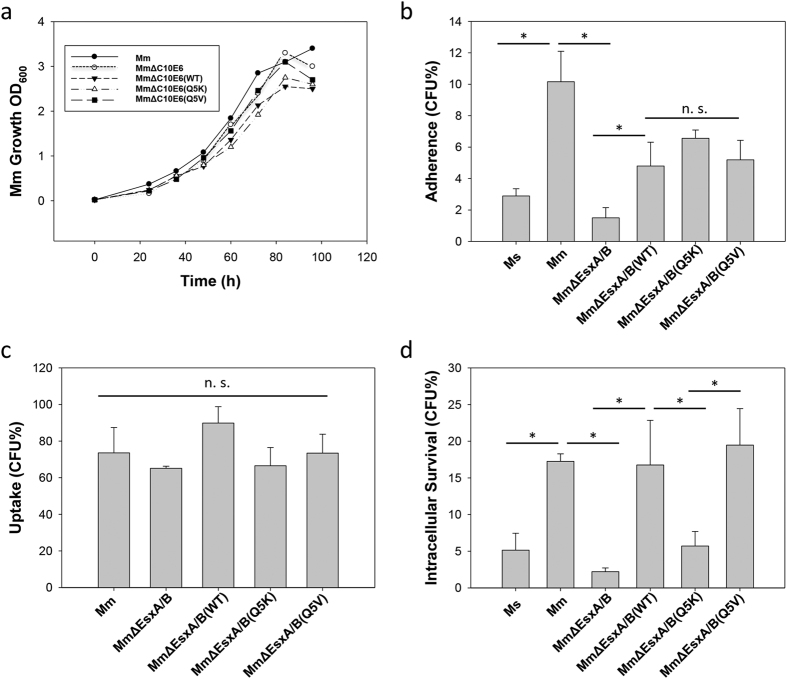
Effects of Q5K and Q5V on mycobacterial growth, adherence, uptake and intracellular survival. (**a**) The growth curves of *Mm, Mm*ΔEsxA/B, *Mm*ΔEsxA/B(WT), *Mm*ΔEsxA/B(Q5K) and *Mm*ΔEsxA/B(Q5V) *in vitro*. The indicated mycobacteria with same concentration were grown in 7H9 medium at 30 **°**C. The concentrations of mycobacteria at the indicated times were measured with OD_600_. The represented data from three independent experiments were shown. (**b**) Mycobacterial adherence to RAW264.7 cells. Prior to test, Raw264.7 cells were fixed by 4% PFA. Single-cell suspensions of mycobacteria were incubated with the fixed cells at MOI 10:1 for 30 min. The free mycobacteria were removed by 3 washes with PBS. The cells were lysed and plated on 7H10 plates with appropriate antibiotics. CFU was counted and the adherence (%) was calculated as adhered bacteria/total input (%). The data is presented as mean ± S.E (n = 6, *p < 0.05). (**c**) Mycobacterial uptake by RAW264.7 cells. Single-cell suspensions were incubated with RAW264.7 cells at MOI 10:1 for 2 h. The extracellular mycobacteria were killed by amikacin and washed off with PBS. The cells were lysed and plated on 7H10 plates with appropriate antibiotics. CFU for each strain was counted and the uptake (%) was calculated as engulfed bacteria/adhered bacteria (%). (**d**) Intracellular survival in RAW264.7 cells. After 2 h infection, cells were washed and incubated in fresh medium for 3 days before lysed and plated. Data were calculated as the CFU (after 3 days infection)/the original input (%). The data is presented as mean ± S.E. (n = 6, *p < 0.05).

We set out to test if Q5K and Q5V affect mycobacterial *in vitro* growth, which was monitored by OD_600_ over time. Compared to *Mm, Mm*∆EsxA/B had a similar growth rate, indicating that deletion of EsxA/B does not affect *in vitro* growth. As expected, the complemented WT, Q5K and Q5V strains showed similar *in vitro* growth (Fig. 3a).

Mycobacterial infection of host cells is a multi-step event. To dissect the role of EsxA in mycobacterial infection, we tested the effects of Q5K and Q5V on mycobacterial adherence, uptake and intracellular survival in macrophages. Interestingly, compared to *Ms*, which had little adherence to the pre-fixed RAW264.7 cells, *Mm* had a significantly higher adherence (Fig. 3b). This result is consistent to the previous report^38^. The ability of *Mm* to adhere to macrophages appears to depend on EsxB/EsxA, because *Mm*∆EsxA/B had a greatly reduced adherence, while complementation with EsxB/EsxA largely restored the adherence. More interestingly, there was no significant difference in adherence between the complemented strains *Mm*∆EsxA/B(WT), *Mm*∆EsxA/B(Q5K), and *Mm*∆EsxA/B(Q5V), suggesting that Q5K and Q5V don’t affect adherence (Fig. 3b). Subsequently, the ability of each *Mm* strain to enter macrophages was tested in the live RAW264.7 cells, and the result (CFU) was normalized to the adherence level of each strain (Fig. 3c). All strains showed similar entry efficiency, suggesting that once attached, deletion of EsxA/B or mutations at Q5 do not affect mycobacterial entry into macrophages. Finally, we tested the effects of Q5K and Q5V on mycobacterial intracellular survival. As expected, *Mm* had a much higher intracellular survival than *Ms* and *Mm*∆EsxA/B (Fig. 3d). Notably, *Mm*∆EsxA/B(WT) and *Mm*∆EsxA/B(Q5V) had higher intracellular survival than that of *Mm*∆EsxA/B(Q5K). This data strongly suggests that the membrane-permeabilizing activity of EsxA is essential for mycobacterial intracellular survival.

### Effects of Q5K and Q5V on mycobacterial cytotoxicity

**Figure 4:**
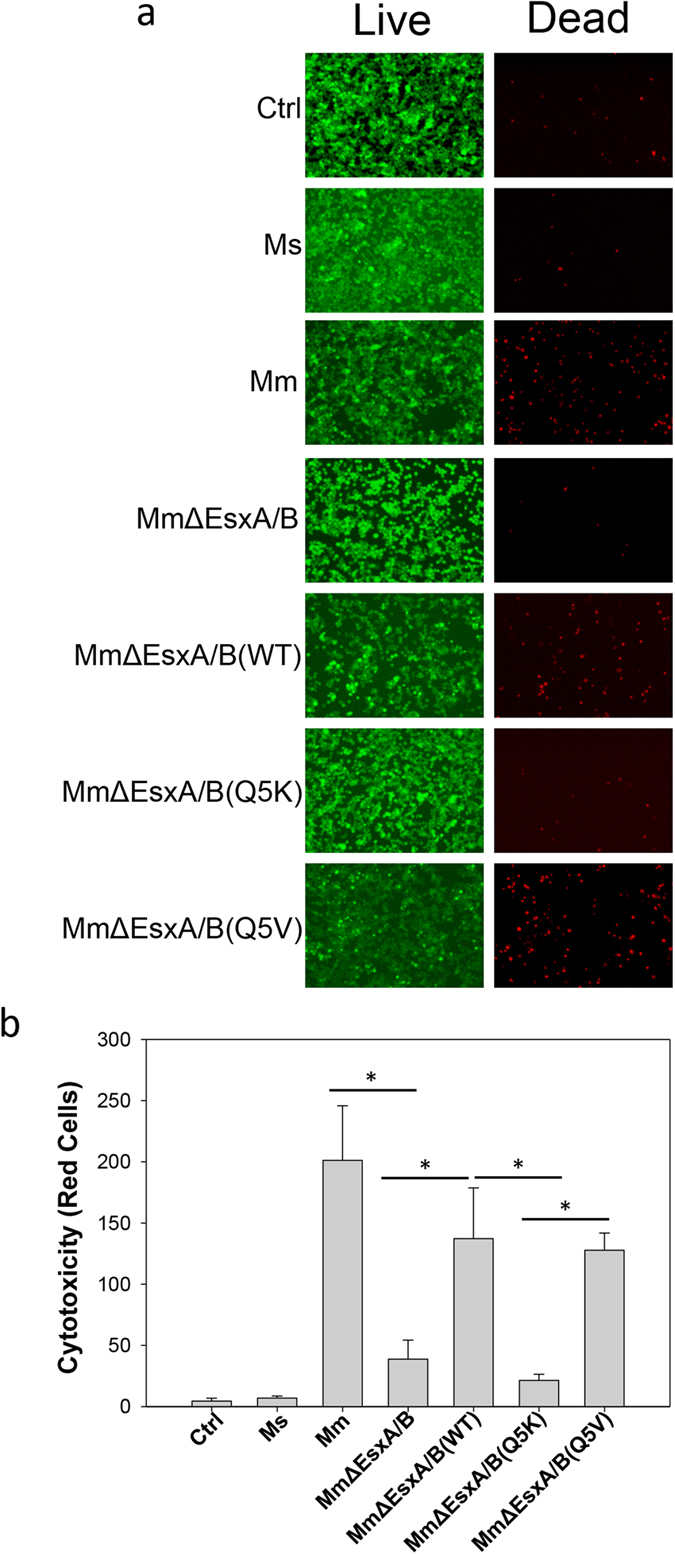
Effects of Q5K and Q5V on mycobacterial cytotoxicity. (**a**) RAW264.7 cells were infected at an MOI of 20 for 3 h. The cells were stained with EthD-1 (label dead cells as red) and Calcein-AM (label live cells as green). Representative images taken in a confocal microscope were shown. (**b**) Quantification of the cytotoxicity. The red cells in 8 random fields for each infection were counted and presented as mean ± S.E. (n = 8, *p < 0.05). Control (Ctrl): mock infection.

Infection is a live-or-perish process between host cells and bacteria. The more intracellular survival of bacteria usually means more host cell death. We further tested the effects of Q5K and Q5V on *mycobacteri*al cytotoxicity in RAW264.7 cells. Here, we used a live/dead assay to stain the infected cells with dead cells in red and live cells in green. As expected, *Ms* showed little cytotoxic effect on the cells, while *Mm* caused cytotoxicity that was significantly higher than *Mm*∆EsxA/B (Fig. 4). Among the complemented strains, *Mm*∆EsxA/B(WT) and *Mm*∆EsxA/B(Q5V) induced cytotoxicity that was much higher than *Mm*∆EsxA/B(Q5K) (Fig. 4). The result of cytotoxicity is well consistent to the result of intracellular survival. Both assays have clearly demonstrated that the membrane-permeabilizing activity of EsxA is an essential factor for mycobacterial intracellular survival and cytotoxicity.

### Effects of Q5K and Q5V on mycobacterial cytosolic translocation

**Figure 5:**
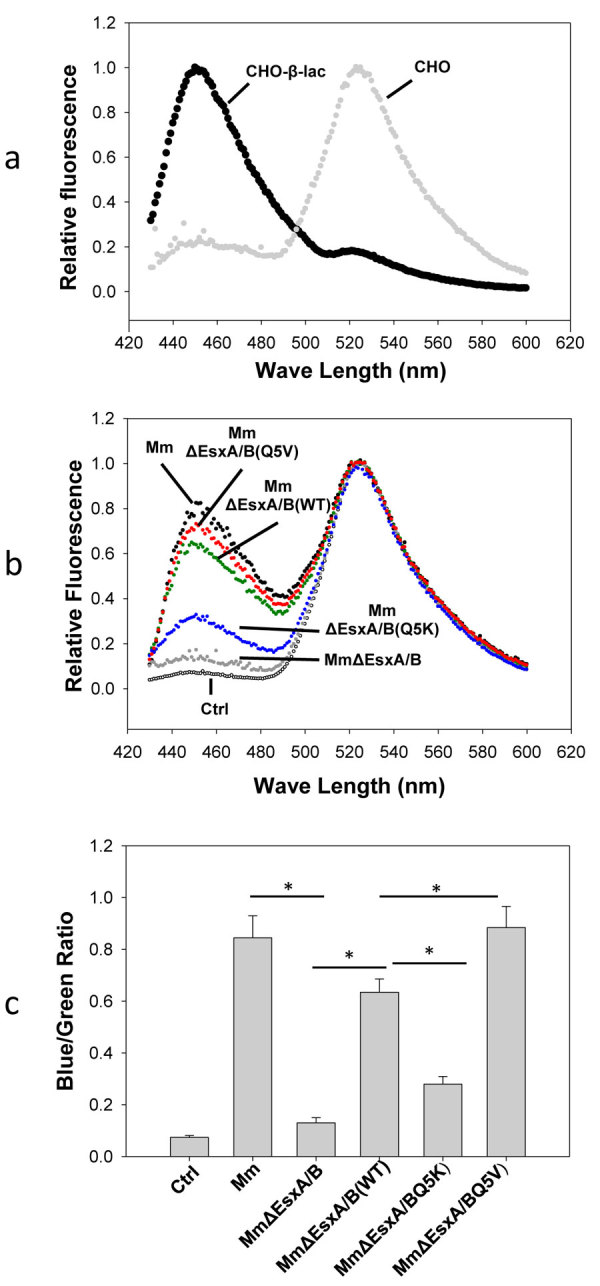
Effects of Q5K and Q5V on cytosolic translocation of *Mm*. (**a**) CHO cells and CHO-β-lac cells were loaded with CCF4-AM, and CCF4 FRET was monitored at emission 425–600 nm with excitation at 409 nm. (**b**) RAW264.7 cells were infected with *Mm, Mm*ΔEsxA/B, *Mm*ΔEsxA/B(WT), *Mm*ΔEsxA/B(Q5K) and *Mm*ΔEsxA/B(Q5V) for 2 h. The infected cells were further incubated for 48 h at 30 °C and then loaded with CCF4-AM. Emission spectra of CCF4 were monitored at 425–600 nm, with excitation at 409 nm. (**c**) Blue/Green ratios in the RAW264.7 infected by the indicated mycobacteria were calculated as I_450_/I_530_. The data was obtained from three independent experiments and were presented as mean ± S.E. (n = 3, *p < 0.05). Control (Ctrl): mock infection.

We hypothesized that the membrane-permeabilizing activity of EsxA contributes to mycobacterial intracellular survival and cytotoxicity through catalyzing translocation of mycobacteria from the phagosome to the cytosol. To probe mycobacterial cytosolic translocation, we used CCF4-AM, a well-established bacterial β-lactamase sensitive FRET reporter^8^,^39^,^40^,^41^. CCF4-AM is composed of a fluorescence donor (7-hydroxycoumarin, blue), a fluorescence acceptor (fluorescein, green) as well as a cephalosporin core, which is the cleavable substrate of bacterial β-lactamase. When mycobacteria rupture the phagosomal membrane and replicate in the cytosol, mycobacterial β-lactamase cleaves CCF4, which separates the fluorescence donor and acceptor and results in loss of FRET signal (shift from green to blue). To ensure timely, accurate quantification of FRET signal change from the infected RAW264.7 cells, we used an ISS-K2 fluorometer, which allows us to monitor the overall change of FRET signal from a large number of cells in a single measurement. To validate the approach, we first used the CHO (Chinese hamster ovary) cell line that stably expresses β-lactamase (CHO-β-lac) in comparison with the parental CHO cell line (Fig. 5a). We found that with excitation at 409 nm, CHO cells had the emission peak at ~530 nm (green). In contrast, CHO-β-lac cells had the emission peak at ~450 nm (blue), indicating that CCF4 is cleaved by β-lactamase. Thus, the K2 fluorometer is suitable to measure CCF4-FRET signal in cells. Next, we measured the FRET changes (blue/green ratio) in the RAW264.7 cells infected by the mycobacterial strains. As shown in Fig. 5b,c, the blue/green ratio of the *Mm*-infected cells is significantly higher than that of the *Mm*∆EsxA/B-infected cells, indicating EsxA/B is required for cytosolic translocation. In the infection by the complemented strains, the cells infected by *Mm*ΔEsxA/B(Q5V) had the highest blue/green ratio, which was followed by *Mm*ΔEsxA/B(WT) and by *Mm*ΔEsxA/B(Q5K), The phenotype of cytosolic translocation is correlated very well to the membrane-permeabilizing activity.

### Effects of Q5K and Q5V on mycobacterial infection in zebra fish embryos

**Figure 6:**
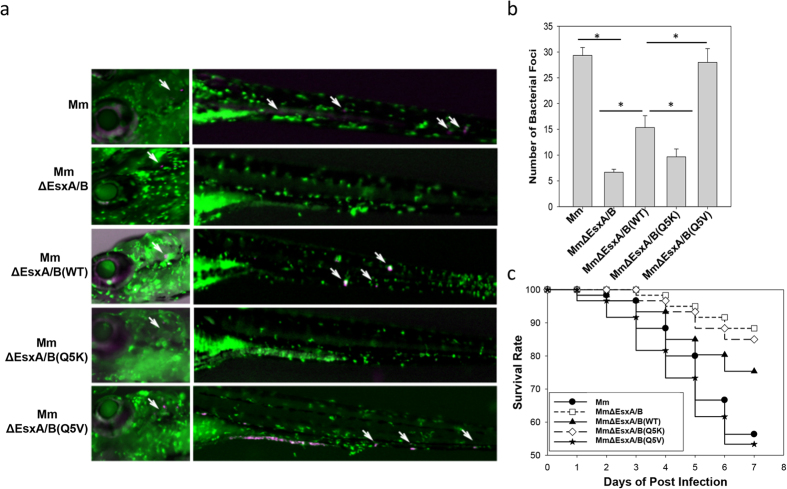
Effects of Q5K and Q5V on *Mm* infection of zebra fish embryos. (**a**) Mycobacterial dissemination. The zebra fish embryos at the day 3 of post-fertilization were infected by the indicated *Mm* strains through ear injection (1000 CFU/fish). At the day 3 of post-infection, the embryos were observed microscopically and mycobacterial dissemination was recorded. The injection sites on the heads and the disseminated mycobacteria (mCherry) engulfed by macrophages (green) were labeled by arrows. (**b**) The numbers of disseminated mycobacterial foci in the fishes were calculated from three independent experiments. The data is presented as mean ± S.E (n = 60, *p < 0.05). (**c**) Fish survival plot. Death of embryos was monitored everyday after infection. Twenty fishes were tested for each group and the experiment was repeated three independent times. The survival rate (%) was calculated and plotted against days of post-infection.

To test the effects of Q5K and Q5V on mycobacterial infection *in vivo*, we used zebra fish as an animal model^34^,^42^. The zebra fish are engineered to express a green fluorescent protein, Dendra2, in macrophages^43^. We also expressed a mCherry protein in the mycobacterial strains so that bacteria can be also tracked inside the fish. The fish embryos at the day 3 of post-fertilization were infected by the indicated strains through ear injection and were examined under a microscope everyday after infection (Fig. 6a,b). At the day 3 of post-infection (DPI), a significant number of *Mm* foci were found at the remote sites of the fish bodies, indicating that *Mm* disseminated in the fish. On the contrary, we found few *Mm*ΔEsxA/B foci disseminated from the injection site. As expected, the number of *Mm*ΔEsxA/B(Q5V) foci at the remote sites is significantly higher than that of *Mm*ΔEsxA/B(WT), and the number of *Mm*ΔEsxA/B(WT) foci is significantly higher than that of *Mm*ΔEsxA/B(Q5K) (Fig. 6b). In parallel to dissemination experiment, we calculated the survival rate up to 7 DPI. Consistent to the dissemination data, *Mm*-infected embryos had ~55% survival at 7 DPI, while *Mm*ΔEsxA/B-infected embryos still had ~88% survival. Within the complemented strains, *Mm*ΔEsxA/B(Q5V) caused the highest death (~53% survival), which was followed by *Mm*ΔEsxA/B(WT) (~74% survival) and by *Mm*ΔEsxA/B(Q5K) (86% survival) with significant difference (Fig. 6c) (Table S1).

### Effects of Q5K and Q5V on *Mtb*-induced cytotoxicity in THP-1 cells

**Figure 7:**
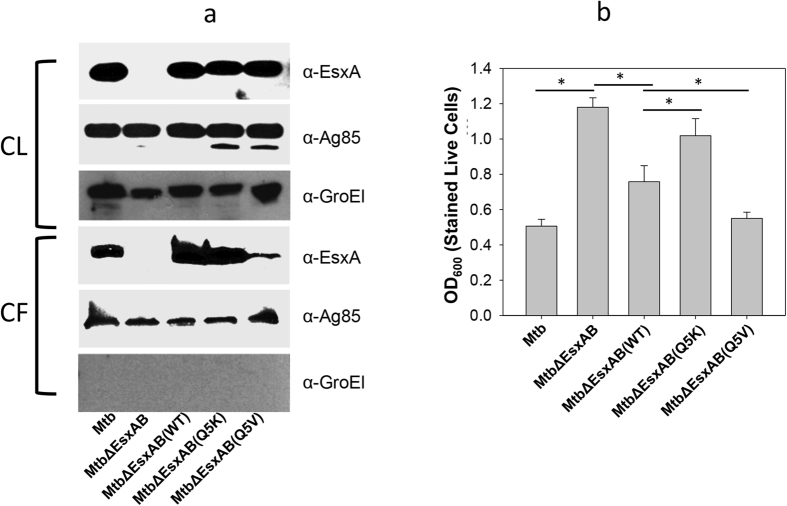
Effects of Q5K and Q5V on *Mtb* cytotoxicity in THP-1 cells. (**a)** Expression and secretion of EsxA protein in *Mtb*. The cell lysate (CL) and culture filtrate (CF) of the indicated *Mtb* strains were loaded to SDS-PAGE, followed by western blotting using the indicated antibodies. The secreted antigen Ag85 was used as a loading control in both CL and CF. GroEl was used to control the potential cross-contamination between CF and CL. (**b**) THP-1 cells were infected with the indicated strains for 72 h at MOI of 10:1. The attached live cells were stained with crystal violet and measured as OD_600_. The data were presented as mean ± S.E (n = 4, *p < 0.05).

Finally, we tested whether the findings made in *Mm* can be replicated in *Mtb* infection in human macrophages. Similarly, we generated the complemented *Mtb* strains by transferring pMH406(WT), pMH406(Q5K) and pMH406(Q5K) into *Mtb*ΔEsxA/B. Expression and secretion of the *Mtb*EsxA WT, Q5K and Q5V were detected by western blot (Fig. 7a). Interestingly, we noticed that there was much less Q5V protein in the culture filtrate relative to other proteins (Fig. 7b). To measure the *Mtb*-induced cytotoxicity, we used the crystal violet uptake assay, which is a colorimetric method that quantifies the cytotoxic effect as a function of the remaining live cells after infection^44^. The reason why we quantified live cells instead of dead cells as described in Live/Dead assay (Fig. 4) was because that a large number of dead cells were washed off the plates after a long-period of infection, which undercounted dead cells (cytotoxicity), especially for the virulent strains. As expected, *Mtb* had the least remaining cells (OD_600_ ~0.5), while *Mtb*ΔEsxA/B had the most remaining cells (OD_600_ ~ 1.2). Within the complemented strains, *Mm*ΔEsxA/B(Q5V) was the most cytotoxic (OD_600_ ~ 0.52), which was followed by *Mm*ΔEsxA/B(WT) (~0.75) and by *Mm*ΔEsxA/B(Q5K) (~1.1) (Fig. 7b).

### Effects of Q5K and Q5V on cytosolic translocation of *Mtb*

**Figure 8:**
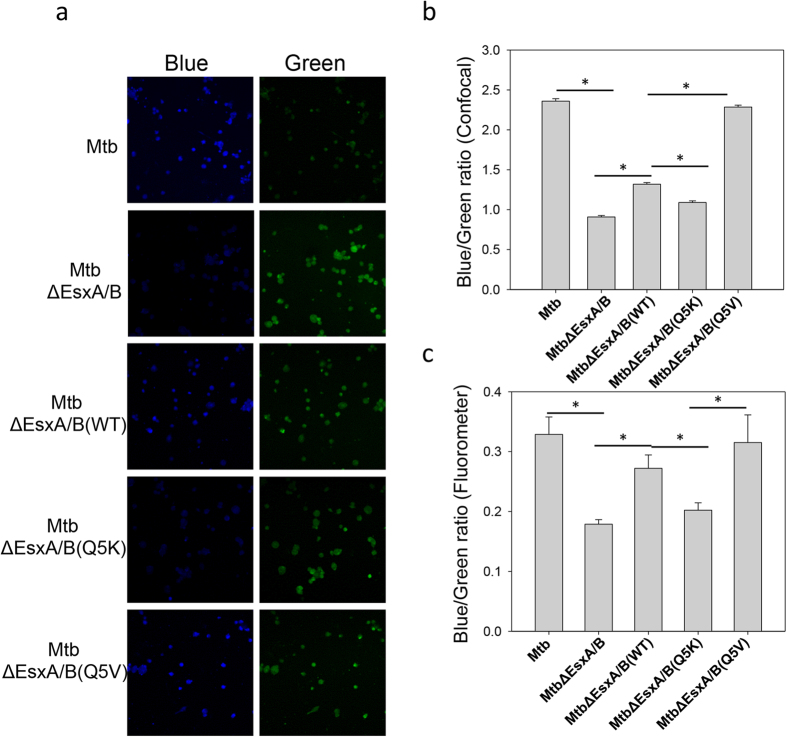
Effects of Q5K and Q5V on cytosolic translocation of *Mtb*. (**a**) THP-1 cells were infected with the indicated *Mtb* strains at a MOI of 2 for 6 days and then loaded with CCF4-AM for 2 h. The cells were imaged at both blue and green channels in a confocal fluorescence microscope. (**b**) Blue/green ratio of each cell in the random fields was measured by Cell Prolifer 2.0. The data were calculated from at least 1000 cells for each infection (*p < 0.05). (**c**) THP-1 cells were infected with the indicated *Mtb* strains at a MOI of 2 for 9 days and then loaded with CCF4-AM for 2 h. The cells were washed, detached and fixed by PFA. Blue (450 nm)/green (530 nm) ratio was measured in the fluorometer with excitation at 409 nm. The data is presented as mean ± S.E. (n = 3, *p < 0.05).

Phagosome rupture and cytosolic translocation of *Mtb* in THP-1 cells were tested using CCF4-AM FRET by two independent approaches: confocal fluorescence microscopy (Fig. 8a,b) and emission spectra in the fluorometer (Fig. 8c). Consistent to the results obtained in *Mm*, the blue/green ratio of *Mtb*-infected cells is significantly higher than that of *Mtb*ΔEsxA/B. Among the complemented strains, the *Mtb*ΔEsxA/B(Q5V)-infected cells had the highest blue/green ratio, which was followed by *Mtb*ΔEsxA/B(WT) and by *Mtb*ΔEsxA/B(Q5K).

## Discussion

While current studies strongly suggest that EsxA is required for virulence of mycobacteria, particularly through facilitating mycobacterial phagosome-to-cytosol translocation^6^,^7^,^8^,^11^, direct evidence is missing that the membrane-permeabilizing activity of EsxA catalyzes the critical pathogenic process. In the present study, we unambiguously demonstrated that Q5V and Q5K either up or down regulated the membrane-permeabilizing activity of EsxA, and consequently up or down regulated mycobacterial cytosolic translocation and virulence both in cultured macrophages and in zebra fish. As one of the most successful bacterial pathogens, *Mtb* has been reported to establish infection by using multiple virulence factors^45^. Thus, it is surprising that single-residue mutations of a single virulence factor caused such a dramatic effect on the virulence. The results strongly argue that the EsxA-catalyzed phagosome-to-cytosol translation is one of the major mechanisms in mycobacterial pathogenesis. Thus, the membrane-permeabilizing activity of EsxA can be a promising therapeutic target.

It is an interesting finding that various mutations of Q5 either up or down regulated the membrane-permeabilizing activity of EsxA, which was apparently affected by the degree of charge state or hydrophobicity at this position upon acidification. Since the structure of the putative EsxA pore is not available, the mechanism underlying this observation is not clear. Our earlier study has shown that the N- and C-terminal flexible arms are required for pore formation, but they do not physically insert into the membrane, instead they may function in attaching EsxA to the membrane surface and supporting membrane insertion of the central Helix-turn-Helix motif 30. Moreover, EsxA pore is likely to be an oligomeric complex in the membrane. Thus, the N- and C-terminal flexible arms may be involved in intra- and/or inter-molecular interactions within the complex to facilitate pore formation. One can imagine that mutations at Q5 could regulate membrane-permeabilizting activity through any of the potential mechanisms described above.

Recently, we have found that the differential membrane-permeabilizing activity between *Mtb*EsxA and MsEsxA is at least partly attributed to the residues at position 25–26, where *Mtb*EsxA has I-H, which serve a pH sensor, while *Ms*EsxA has T-A. Exchange of IH and TA completely restored *Ms*EsxA membrane-permeabilizing activity to a level equivalent to *Mtb*EsxA, but only partly affected *Mtb*EsxA^46^. Because V is on *Ms*EsxA, we initially predicted that replacing Q5 with V would decrease the membrane-permeabilizing activity of *Mtb*EsxA. The results in this study made us to believe that besides Q5 and I25H26, there are other residues responsible for the differential membrane-permeabilizing activity between *Mtb*EsxA and *Ms*EsxA, which warrants further investigation into the mechanism of EsxA membrane permeabilization.

Relative to the complemented WT strain, the complemented Q5V exhibited significantly stronger virulence in zebra fish embryos and in THP-1 infection measured by crystal violet uptake assay (Fig. 6, Fig. 7b). However, Q5V showed intracellular survival (Fig. 3d) and cytotoxicity (by Live/Dead assay) that were similar to the complemented WT (Fig. 4). This discrepancy is mainly due to the loss of dead cells during washes after infection. This is especially true for the cells infected by the virulent strains. The more virulent, the more cell loss. Thus, the number of dead cells (e.g. Fig. 4) is usually undercounted for the virulent strains. CCF-4 FRET assay is a quantitative approach that measures blue/green ratio for each individual cell, and it is more independent of the number of remaining cells on the plates. Thus, Q5V showed significantly higher cytosolic translocation in most CCF-4 FRET assays (Fig. 5c, Fig. 8b). Finally, we observed that the amount of Q5V protein in culture filtrate of *Mtb*ΔEsxA/B(Q5V) was lower than others (Fig. 7a), suggesting that Q5V may have a lower secretion in *Mtb*. The exact mechanism is not known and may be investigated in future study. Even though the secretion of Q5V protein was lower, *Mtb*ΔEsxA/B(Q5V) still exhibited stronger cytotoxicity (Fig. 7b) and cytosolic translocation (Fig. 8) than *Mtb*ΔEsxA/B(WT), which further supports our conclusion that Q5V enhanced virulence through increasing membrane-permeabilizing activity.

Another interesting observation is that *Mm*ΔEsxA/B failed to adhere to the fixed RAW264.7 cells. To the best of our knowledge, this is the first report that EsxA is required for mycobacterial adherence to macrophages. While only ~10% of *Mm* was found to be adhered to the fixed cells, which is consistent to the previous study by another group^38^, it represented a large number of mycobacteria that provided us a big enough sample size for analysis of statistical significance. More importantly, all of strains used in the experiments, including *Ms, Mm, Mm*ΔEsxA/B, *Mm*ΔEsxA/B(WT), *Mm*ΔEsxA/B(Q5K), and *Mm*ΔEsxA/B(Q5V), were excellent internal controls to each other, which demonstrated the specificity of the results. It is generally accepted that EsxA is a secreted protein, but it has been reported that *Mm* and *Mtb* with deficient EsxA exhibited different colony morphologies, implicating that EsxA binds to mycobacterial cell walls^47^. Recently, Champion *et al*. showed that some EsxA molecules remained bound to the surface of mycobacteria instead of being secreted into the medium. Moreover, EsxA on the surface of mycobacteria caused more cytotoxicity than those secreted^48^,^49^. There are also reports that EsxA binds to the host cells through interacting with specific receptor molecules on the cell surface^50^,^51^,^52^. In zebra fish, ESX-1/EsxA has been implicated in recruitment of macrophages to granuloma^53^,^54^. Together, all the evidence suggests that EsxA may play a role in adhering mycobacteria to host cells, which requires further investigation. Since Q5K and Q5V did not affect adherence (Fig. 3b), suggesting that adherence is independent of EsxA membrane-permeabilizing activity.

This study has directly linked the membrane-permeabilizing activity of EsxA to mycobacterial cytosolic translocation, intracellular survival and cytotoxicity. It clearly supports the model that EsxA membrane-permeabilizing activity catalyzes phagosome rupture and mycobacterial translocation to the cytosol, where mycobacteria replicate and undergo cell-to-cell spreading. Since the structure of the putative EsxA pore is not available, the molecular mechanism by which EsxA causes phagosome rupture is largely unknown. It is possible that other mycobacterial and/or host factors may be also involved in this process^55^. It is well known that EsxB and EsxA form a heterodimer^25^,^26^, but EsxB does not have membrane-lytic activity and is considered as a putative chaperone that protects EsxA from being degraded or prevents pre-matured membrane permeabilization by EsxA^31^,^33^. Using surface plasmon resonance, de Jonge *et al*. showed that CFP10 was dissociated from EsxA in an acidic pH-dependent manner^31^. In our earlier pH titration experiment, EsxA only formed pores on the liposomal membrane at pH 5 or below^33^. All the data argue that an acidic pH is required for EsxA to permeabilize the membrane. However, recent studies also suggest that EsxA plays a role in arresting phagosome maturation, where most intracellular mycobacteria stayed in poorly acidified phagosomes, while ESX-1 knockout strains stayed in acidified phagosomes^9^,^10^. Most recently, Simeone *et al*. showed that partial prevention of phagosome acidification is a prerequisite for mycobacterial phagosome rupture and cytosolic access^11^. This discrepancy may be due to the difference between *in vitro* and *in vivo* experimental conditions, where *in vivo* mycobacterial and/or host factors may allow EsxA to form pores at a higher pH. Another possibility is that the two events may actually occur at different stages of infection. That is, EsxA may inhibit phagosome maturation at early stage of infection to establish latency, while at the later stage of infection EsxA catalyzes mycobacterial escape from the phagosome. An earlier report has shown that upon lysosome-phagosome fusion, *Mtb* progressively translocates from phago-lysosomes into the cytosol^6^. Therefore, one can imagine that phagosome-lysosome fusion may be a trigger for mycobacteria to escape from the phagosome to the cytosol. Upon phagosome-lysosome fusion, acidification induces EsxA-mediated membrane permeabilization, which allows mycobacteria escape to the cytosol before being killed by the hostile environment.

In addition to catalyzing mycobacterial cytosolic translocation, several independent studies have suggested that even prior to full-fledged phagosomal rupture, EsxA may permeabilize the phagosomal membrane to expose mycobacterial DNA to the host cytosolic DNA-sensing pathway, which results in a series of host anti-mycobacterial immune responses, including induction of autophagy and cytokine release^56^,^57^,^58^,^59^,^60^,^61^,^62^.

As a potent T-cell antigen, EsxA has been intensively studied as a potential candidate for novel TB vaccines^63^. However, the membrane-permeabilizing activity raises a safety concern when EsxA WT is used as a vaccine. The single-point mutations at Q5, such as Q5K or Q5R, reduce cytotoxicity, but still possibly maintain antigenicity. Thus, they have potentials to be developed into safer vaccines and therapeutics against tuberculosis.

## Experimental Procedures

### Protein purification

*Mtb*ESAT6 WT, Q5E, Q5V, and Q5L were cloned into pET22b vector and expressed and purified from the inclusion body of *E. coli* BL21(DE3) as previously described^33^. Q5R and Q5K had little expression in pET22b vector, so they were sub-cloned into pGEX4T-1 to be expressed as fusion proteins with a N-terminal GST and a C-terminal His-tag. The GST-EsxA fusion proteins were expressed and purified as previously described^30^,^33^.

### ANTS/DPX Membrane leakage assay

The liposomes containing the dye/quencher pair, 8-aminonapthalene-1,3,6- trisulfonic acid (ANTS)/*p*-xylene-bis-pyridinium bromide (DPX), were prepared by rehydrating the dry lipid film in 50 mM ANTS, 50 mM DPX, 5 mM HEPES (pH 7.3), followed by extrusion through a 200-nm filter and by desalting in a G-25 column in 5 mM HEPES, 150 mM NaCl (pH 7.3).

ANTS fluorescence dequenching was measured in an ISS K2 multiphase frequency and modulation fluorometer with excitation 380 nm and emission at 520 nm as described previously^30^,^33^. Briefly, 100 μl of the liposomes containing ANTS/DPX was diluted into 1.3 ml of 50 mM sodium acetate and 150 mM NaCl (pH 4.0) with continuous stirring. After the baseline was stabilized, 100 μl of purified proteins (total of 100 μg) was injected into the cuvette, and the fluorescence signal was monitored in real time.

### Membrane insertion assay by NBD

EsxA(S35C) and EsxA(Q5K/S35C) were purified and then labeled with IANBD (Invitrogen) as described previously^30^,^64^,^65^. The labeling efficiency is ~100%. The NBD-labeled proteins (80 μg) were incubated in 20 mM TrisHCl, 100 mM NaCl (pH 7.0) with or without liposomes for 30 min, and were transferred to a cuvette with a stirring bar in the ISS K2 fluorometer. Acidification was triggered by adding 0.1 volume of 1 M NaAc (pH 4.0) to the cuvette. NBD was excited at 488 nm, and emission was recorded at 544 nm. Crossed polarizers on excitation and emission beams and a 520-nm-long filter were used to reduce the background scatter.

### Cell culture

RAW264.7 cells (ATCC) were maintained in DMEM medium supplemented with 10% NCBS in 5% CO_2_ at 37 °C. THP-1 cells (ATCC) were maintained in RPMI 1640 supplemented with 10% FBS in 5% CO_2_ at 37 °C. Before experiments, THP-1 cells were induced with 20 ng/ml of phorbol 12-myristate 13-acetate (PMA) for 72 h to differentiate into macrophage-like cells. Chinese hamster ovary (CHO) cells and CHO-β-Lac cells (Life Technologies Inc.) were maintained in F-12 medium supplemented with 10% NBS in 5% CO_2_ at 37 °C.

### Construction esxB/esxA knockout Mm strain

The knockout strain *Mm*ΔEsxA/B was generated by homologous recombination with modifications^36^. The fragments at left arm and right arm of the *esxB/esxA* operon were amplified from *Mm* genomic DNA using the primers P_LA_-1/P_LA_-2 and P_RA_-1/P_RA_-2 (Table S2). The PCR products of left arm and right arm were then cloned into the pJSC407 plasmid (generous gift from Dr. Jeffery Cox, UCSF) using ligation-free PCR cloning kit (Clontech). The resultant plasmid was digested by *Pac* I and ligated to a sucrose resistance gene from pGOAL17 (Addgen). The resultant plasmid was then introduced into *Mm* by electroporation. Single-crossover clones were first selected on 7H10 plates supplemented with 10% OADC and containing 50 μg/ml hygromycin, followed by counterselection of double-crossover clones on 7H10 plates containing 2% sucrose plates. Deletion of *esxB*/*esxA* was confirmed by PCR and western blot. The *hyg*^*r*^ gene was later removed using Cre recombinase.

### Complementation of *Mm*ΔEsxA/B and MtbΔEsxA/B

The plasmids pMH406 constitutively expresses *Mtb*EsxB and *Mtb*EsxA in mycobacteria^18^. The plasmids pMH406(WT), pMH406(Q5K) and pMH406(Q5V) were electroporated into *Mm*ΔEsxA/B to generate the complemented strains, named *Mm*ΔEsxA/B(WT), *Mm*ΔEsxA/B(Q5K) and *Mm*ΔEsxA/B(Q5V), respectively.

Similarly, pMH406(WT), pMH406(Q5K) and pMH406(Q5V) were transferred into *Mtb*ΔEsxA/B to generate *Mtb*ΔEsxA/B(WT), *Mtb*ΔEsxA/B(Q5K) and pMH406(Q5V).

### Mycobacterial adherence, uptake and intracellular survival

Mycobacterial adherence were carried out as previously described with modifications^38^. Briefly, RAW264.7 (0.5 × 10^6^ cells/well) were fixed in 4% paraformaldehyde (PFA) for 10 min. Mycobacteria were prepared as single-cell solution as previously described^66^, and then added to the cells with a multiplicity of infection (MOI) of 10. After 30 min of incubation, the free mycobacteria were removed by 3 washes with PBS. The bound mycobacteria were harvested and plated onto 7H10 plates to determine colony-forming units (CFU). The rate of adherence was calculated as adhered bacteria/total input (%).

In the uptake assay the mycobacteria were incubated with live RAW264.7 cells for 2 h at 37 °C. The extracellular mycobacteria were removed by washes and amikacin. The cells were then lysed in 0.1% Triton X-100 and plated on 7H10 plates to determine CFU of the intracellular mycobacteria. The rate of uptake was calculated as engulfed bacteria/the adhered (%).

In intracellular survival assay, the infection was same as the uptake assay. The infected cells were maintained in growth medium for 3 days before lysed and plated. The rate of intracellular survival is expressed as the CFU after 3 days of infection/the CFU of adherence (%).

### Western blot analysis

*Mm* or *Mtb* grown in Sauton’s medium were harvested, and the supernatant was filtered through a 0.22 μm filter twice to generate culture filtrate (CF). The CF was concentrated by 100 folds as described previously^36^. The whole cell lysate (CL) was obtained by lysing mycobacterial cells in a bead beater.

20 μg of CL and equivalent amount of CF were separated in 12% Tricine-SDS gels. Proteins were transferred to PVDF membrane and subjected to western blot using antibodies against EsxA (AB26246; Abcam, Cambridge, MA), EsxB (NR-13801; BEI Resources, Manassas, VA). GroEL (NR13813, BEI) and Ag85 (NR-13807; BEI) were used as loading controls for CL and CF fractions.

### Mycobacteria-induced cytotoxicity

Live/dead assay for the *Mm*–infected RAW264.7 cells–RAW264.7 cells (5 × 10^5^) were infected with the indicated *Mm* strains at an MOI of 20 for 2 h at 37 °C, followed by another 3 h incubation after washing off the free bacteria. The infected cells were stained with ethidium homodimer and calcein-AM (Life Technologies) at 37 °C for 1 h, and images were taken in a Floid Cell Imaging Station. The relative cytotoxicity was quantified by enumeration of dead cells in at least 6 random fields for each infection.

Crystal violet uptake assay for the *Mtb-infected* THP-1 cells–The PMA-differentiated THP-1 cells (5 × 10^5^) were infected with the indicated *Mtb* strains at an MOI of 10 for 72 h at 37 °C and fixed with 4% PFA for 30 min, and then stained with 0.1% aqueous crystal violet for 30 min with gentle shaking. The excessive dye was washed off with water. The plates were allowed to air dry and the stained cells were lysed in 0.2% Triton X-100 for 30 min at RT with gentle shaking. The supernatant was transferred into a 96-well plate for OD_600_ measurement.

### Mycobacterial cytosolic translocation

For *Mm* strains, RAW264.7 cells (3 × 10^6^) were infected with single-cell solutions of the indicated *Mm* strains at MOI of 10 for 2 h at 30 °C, followed by another 48 h incubation after removing free mycobacteria. The cells were harvested by centrifugation and incubated with 1 μM CCF4-AM for 2 h in dark by following the manufacturer’s instruction (Liveblazer FRET B/G loading kit, Life Technology). After washing off the free CCF4-AM, the cells were incubated in 2 ml of EM buffer with 2.5 μM probenecid acid. The spectrum of CCF4 was measured in the ISS-K2 fluorometer with excitation at 409 nm and emission from 425–600 nm. The blue/green ratio was calculated as the ratio of I_450_/I_530_.

For *Mtb* strains, the PMA-induced THP-cells (1.5 × 10^6^) were infected by the single-cell solutions of the indicated *Mtb* stains at MOI of 3 for 2 h. After removing the free bacteria, the cells was continuously incubated at 37 °C for 7–9 days. We found that at 9 days of post-infection, Mtb and Mtb∆EsxA/B started to show significant difference in blue/green ratio (Figure S1). The cells were loaded with 2 μM CCF4-AM at RT for 2 h. After washing off the free dye, the cells were harvested and fixed by 4% PFA in the presence of 2.5 μM probenecid acid. The blue/green ratio were measured in the ISS-K2 fluorometer and calculated as described above.

For fluorescence microscopy, THP-1 cells were seeded in a 96-well plate (3 × 10^4^ cells/well) and the infection was exactly same as above described except with MOI of 2. After 6 days of infection, the cells were loaded with CCF4-AM as described above. The CCF4 fluorescence was measured in a Zeiss LSM 700 inverted confocal microscope. Blue (450 nm) and green (520 nm) fluorescent pictures were simultaneously taken at the excitation laser (405 nm). Automated image analysis including cell segmentation and quantification was achieved using Cell Prolifer.

### Fish husbandry and embryo infection

The transgenic zebra fish line *Tg(mpeg:dendra2)* was obtained from Dr. Anna Huttenlocher’s lab (UW-Madison, WI, USA). The zebra fish embryos from *Tg(mpeg:dendra2)* were used in this experiment^43^. *Mm* strains harboring a pMSP12::mCherry plasmid (Addgene) were cultured and prepared into single-cell solution with sterile PBS. The embryos were maintained and infected at the day 3 of post-fertilization. About 1000 CFU of mycobacteria were injected in the embryos’ ears. After infection each embryo was housed separately in a 96-well plate. The death of each group (total 20 embryos) was recorded everyday after infection. At the day 3 of post-infection, mycobacterial dissemination was detected with a Zeiss ZV16 stereo-microscope. The infection experiment was repeated three times.

### Statistical analysis

The data were presented as mean ± S.E. One-way ANOVA were used for analysis of statistical significance in SigmaPlot V10 (Systat Software Inc, CA). *P* < 0.05 was considered as statistically significant.

### Ethical approval and informed consent

Approval: The zebra fish experiment was conducted according to the internationally accepted standards. The Animal Care and Use Protocol was approved by The Purdue Animal Care and Use Committee (PACUC), adhering to the Guidelines for Use of Zebra fish in the NIH Intramural Research Program (Protocol number: 1401001018).

Accordance: The methods were carried out in accordance with the approved guidelines.

Informed consent: N/A

## Additional Information

**How to cite this article**: Zhang, Q. *et al*. EsxA membrane-permeabilizing activity plays a key role in mycobacterial cytosolic translocation and virulence: effects of single-residue mutations at glutamine 5. *Sci. Rep.* **6**, 32618; doi: 10.1038/srep32618 (2016).

## Supplementary Material

Supplementary Information

## References

[b1] World Health Organization. Global tuberculosis report 2013 (World Health Organization, 2013).

[b2] Zumla A., Raviglione M., Hafner R. & Fordham von Reyn C. Tuberculosis. N. Engl. J. Med. 368, 745–755 (2013). 23425167 10.1056/NEJMra1200894

[b3] Orme I. Adaptive immunity to mycobacteria. Curr Opin Microbiol 7, 58–61 (2004). 15036141 10.1016/j.mib.2003.11.002

[b4] Kang P. B. . The human macrophage mannose receptor directs Mycobacterium tuberculosis lipoarabinomannan-mediated phagosome biogenesis. J Exp Med 202, 987–999 (2005). 16203868 10.1084/jem.20051239 PMC2213176

[b5] Pizarro-Cerdá J. & Cossart P. Bacterial adhesion and entry into host cells. Cell 124, 715–727 (2006). 16497583 10.1016/j.cell.2006.02.012

[b6] van der Wel N. . M. tuberculosis and M. leprae translocate from the phagolysosome to the cytosol in myeloid cells. Cell 129, 1287–1298 (2007). 17604718 10.1016/j.cell.2007.05.059

[b7] Houben D. . ESX-1 Mediated Translocation to the Cytosol controls Virulence of Mycobacteria. Cell Microbiol, doi: 10.1111/j.1462-5822.2012.01799.x (2012). 22524898

[b8] Simeone R. . Phagosomal rupture by Mycobacterium tuberculosis results in toxicity and host cell death. PLoS Pathog 8, e1002507 (2012). 22319448 10.1371/journal.ppat.1002507 PMC3271072

[b9] MacGurn J. A. & Cox J. S. A Genetic Screen for Mycobacterium tuberculosis Mutants Defective for Phagosome Maturation Arrest Identifies Components of the ESX-1 Secretion System. Infect Immun 75, 2668–2678 (2007). 17353284 10.1128/IAI.01872-06 PMC1932882

[b10] Tan T., Lee W. L., Alexander D. C., Grinstein S. & Liu J. The ESAT-6/CFP-10 secretion system of Mycobacterium marinum modulates phagosome maturation. Cell Microbiol 8, 1417–1429 (2006). 16922861 10.1111/j.1462-5822.2006.00721.x

[b11] Simeone R. . Cytosolic access of Mycobacterium tuberculosis: critical impact of phagosomal acidification control and demonstration of occurrence *in vivo*. PLoS Pathog 11, e1004650 (2015). 25658322 10.1371/journal.ppat.1004650 PMC4450080

[b12] Mahairas G. G., Sabo P. J., Hickey M. J., Singh D. C. & Stover C. K. Molecular analysis of genetic differences between Mycobacterium bovis BCG and virulent M. bovis. J Bacteriol 178, 1274–1282 (1996). 8631702 10.1128/jb.178.5.1274-1282.1996 PMC177799

[b13] Harboe M., Oettinger T., Wiker H. G., Rosenkrands I. & Andersen P. Evidence for occurrence of the ESAT-6 protein in Mycobacterium tuberculosis and virulent Mycobacterium bovis and for its absence in Mycobacterium bovis BCG. Infect Immun 64, 16–22 (1996). 8557334 10.1128/iai.64.1.16-22.1996 PMC173721

[b14] Wards B. J., de Lisle G. W. & Collins D. M. An esat6 knockout mutant of Mycobacterium bovis produced by homologous recombination will contribute to the development of a live tuberculosis vaccine. Tuber Lung Dis 80, 185–189 (2000). 11052907 10.1054/tuld.2000.0244

[b15] Pym A. S., Brodin P., Brosch R., Huerre M. & Cole S. T. Loss of RD1 contributed to the attenuation of the live tuberculosis vaccines Mycobacterium bovis BCG and Mycobacterium microti. Mol Microbiol 46, 709–717 (2002). 12410828 10.1046/j.1365-2958.2002.03237.x

[b16] Pym A. S. . Recombinant BCG exporting ESAT-6 confers enhanced protection against tuberculosis. Nat Med 9, 533–539 (2003). 12692540 10.1038/nm859

[b17] Hsu T. . The primary mechanism of attenuation of bacillus Calmette-Guerin is a loss of secreted lytic function required for invasion of lung interstitial tissue. Proc Natl Acad Sci USA 100, 12420–12425 (2003). 14557547 10.1073/pnas.1635213100 PMC218773

[b18] Lewis K. N. . Deletion of RD1 from Mycobacterium tuberculosis mimics bacille Calmette-Guérin attenuation. J Infect Dis 187, 117–123 (2003). 12508154 10.1086/345862 PMC1458498

[b19] Sassetti C. M. & Rubin E. J. Genetic requirements for mycobacterial survival during infection. Proc Natl Acad Sci USA 100, 12989–12994 (2003). 14569030 10.1073/pnas.2134250100 PMC240732

[b20] Stanley S. A., Raghavan S., Hwang W. W. & Cox J. S. Acute infection and macrophage subversion by Mycobacterium tuberculosis require a specialized secretion system. Proc Natl Acad Sci USA 100, 13001–13006 (2003). 14557536 10.1073/pnas.2235593100 PMC240734

[b21] Guinn K. M. . Individual RD1-region genes are required for export of ESAT-6/CFP-10 and for virulence of Mycobacterium tuberculosis. Mol Microbiol 51, 359–370 (2004). 14756778 10.1046/j.1365-2958.2003.03844.x PMC1458497

[b22] Behr M. A. . Comparative genomics of BCG vaccines by whole-genome DNA microarray. Science 284, 1520–1523 (1999). 10348738 10.1126/science.284.5419.1520

[b23] Gordon S. V. . Identification of variable regions in the genomes of tubercle bacilli using bacterial artificial chromosome arrays. Mol Microbiol 32, 643–655 (1999). 10320585 10.1046/j.1365-2958.1999.01383.x

[b24] Berthet F. X., Rasmussen P. B., Rosenkrands I., Andersen P. & Gicquel B. A Mycobacterium tuberculosis operon encoding ESAT-6 and a novel low-molecular-mass culture filtrate protein (CFP-10). Microbiology (Reading, Engl) 144 (Pt 11), 3195–3203 (1998). 10.1099/00221287-144-11-3195 9846755

[b25] Fortune S. M. . Mutually dependent secretion of proteins required for mycobacterial virulence. Proc Natl Acad Sci USA 102, 10676–10681 (2005). 16030141 10.1073/pnas.0504922102 PMC1176248

[b26] Atmakuri K. & Fortune S. M. Regulation of protein secretion by … protein secretion? Cell Host Microbe 4, 190–191 (2008). 18779042 10.1016/j.chom.2008.08.009

[b27] Andersen P., Andersen A. B., Sørensen A. L. & Nagai S. Recall of long-lived immunity to Mycobacterium tuberculosis infection in mice. J Immunol 154, 3359–3372 (1995). 7897219

[b28] Sørensen A. L., Nagai S., Houen G., Andersen P. & Andersen A. B. Purification and characterization of a low-molecular-mass T-cell antigen secreted by Mycobacterium tuberculosis. Infect Immun 63, 1710–1717 (1995). 7729876 10.1128/iai.63.5.1710-1717.1995 PMC173214

[b29] Lalvani A., Sridhar S. & Reyn, von, C. F. Tuberculosis vaccines: time to reset the paradigm? Thorax 68, 1092–1094 (2013). 23749816 10.1136/thoraxjnl-2013-203456

[b30] Ma Y., Keil V. & Sun J. Characterization of Mycobacterium tuberculosis EsxA membrane insertion: roles of N- and C-terminal flexible arms and central helix-turn-helix motif. J Biol Chem 290, 7314–7322 (2015). 25645924 10.1074/jbc.M114.622076 PMC4358149

[b31] de Jonge M. I. . ESAT-6 from Mycobacterium tuberculosis dissociates from its putative chaperone CFP-10 under acidic conditions and exhibits membrane-lysing activity. J Bacteriol 189, 6028–6034 (2007). 17557817 10.1128/JB.00469-07 PMC1952024

[b32] Smith J. . Evidence for pore formation in host cell membranes by ESX-1-secreted ESAT-6 and its role in Mycobacterium marinum escape from the vacuole. Infect Immun 76, 5478–5487 (2008). 18852239 10.1128/IAI.00614-08 PMC2583575

[b33] De Leon J. . Mycobacterium tuberculosis ESAT-6 Exhibits a Unique Membrane-interacting Activity That Is Not Found in Its Ortholog from Non-pathogenic Mycobacterium smegmatis. J Biol Chem 287, 44184–44191 (2012). 23150662 10.1074/jbc.M112.420869 PMC3531734

[b34] Tobin D. M. & Ramakrishnan L. Comparative pathogenesis of Mycobacterium marinum and Mycobacterium tuberculosis. Cell Microbiol 10, 1027–1039 (2008). 18298637 10.1111/j.1462-5822.2008.01133.x

[b35] Ramakrishnan L. Looking within the zebrafish to understand the tuberculous granuloma. Adv Exp Med Biol 783, 251–266 (2013). 23468113 10.1007/978-1-4614-6111-1_13

[b36] Gao L.-Y. . A mycobacterial virulence gene cluster extending RD1 is required for cytolysis, bacterial spreading and ESAT-6 secretion. Mol Microbiol 53, 1677–1693 (2004). 15341647 10.1111/j.1365-2958.2004.04261.x

[b37] Stinear T. P. . Insights from the complete genome sequence of Mycobacterium marinum on the evolution of Mycobacterium tuberculosis. Genome Res 18, 729–741 (2008). 18403782 10.1101/gr.075069.107 PMC2336800

[b38] El-Etr S. H., Subbian S., Cirillo S. L. G. & Cirillo J. D. Identification of two Mycobacterium marinum loci that affect interactions with macrophages. Infect Immun 72, 6902–6913 (2004). 15557611 10.1128/IAI.72.12.6902-6913.2004 PMC529147

[b39] Enninga J. & Rosenshine I. Imaging the assembly, structure and activity of type III secretion systems. Cell Microbiol 11, 1462–1470 (2009). 19622097 10.1111/j.1462-5822.2009.01360.x

[b40] Charpentier X. & Oswald E. Identification of the secretion and translocation domain of the enteropathogenic and enterohemorrhagic Escherichia coli effector Cif, using TEM-1 beta-lactamase as a new fluorescence-based reporter. J Bacteriol 186, 5486–5495 (2004). 15292151 10.1128/JB.186.16.5486-5495.2004 PMC490934

[b41] Acosta Y. . Imaging cytosolic translocation of Mycobacteria with two-photon fluorescence resonance energy transfer microscopy. Biomed. Opt. Express 5, 3990 (2014). 25426325 10.1364/BOE.5.003990 PMC4242033

[b42] Torraca V., Masud S., Spaink H. P. & Meijer A. H. Macrophage-pathogen interactions in infectious diseases: new therapeutic insights from the zebrafish host model. Dis Model Mech 7, 785–797 (2014). 24973749 10.1242/dmm.015594 PMC4073269

[b43] Harvie E. A., Green J. M., Neely M. N. & Huttenlocher A. Innate immune response to Streptococcus iniae infection in zebrafish larvae. Infect Immun 81, 110–121 (2013). 23090960 10.1128/IAI.00642-12 PMC3536132

[b44] Castro-Garza J. . Use of a colorimetric assay to measure differences in cytotoxicity of Mycobacterium tuberculosis strains. J. Med. Microbiol. 56, 733–737 (2007). 17510256 10.1099/jmm.0.46915-0

[b45] Forrellad M. A. . Virulence factors of the Mycobacterium tuberculosis complex. 4, 3–66 (2013). 10.4161/viru.22329 PMC3544749 23076359

[b46] Peng X. . Characterization of differential pore-forming activities of ESAT-6 proteins from Mycobacterium tuberculosis and Mycobacterium smegmatis. FEBS Lett 590, 509–519 (2016). 26801203 10.1002/1873-3468.12072 PMC4973571

[b47] Carlsson F., Joshi S. A., Rangell L. & Brown E. J. Polar localization of virulence-related Esx-1 secretion in mycobacteria. PLoS Pathog 5, e1000285 (2009). 19180234 10.1371/journal.ppat.1000285 PMC2628743

[b48] Kennedy G. M., Hooley G. C., Champion M. M., Mba Medie F. & Champion P. A. D. A Novel ESX-1 Locus Reveals that Surface-Associated ESX-1 Substrates Mediate Virulence in Mycobacterium marinum. J Bacteriol 196, 1877–1888 (2014). 24610712 10.1128/JB.01502-14 PMC4011007

[b49] Medie F. M., Champion M. M., Williams E. A. & DiGiuseppe Champion P. A. Homeostasis of N-α terminal acetylation of EsxA correlates with virulence in Mycobacterium marinum. Infect Immun, doi: 10.1128/IAI.02153-14 (2014). PMC4249322 25135684

[b50] Pathak S. K. . Direct extracellular interaction between the early secreted antigen ESAT-6 of Mycobacterium tuberculosis and TLR2 inhibits TLR signaling in macrophages. Nat Immunol 8, 610–618 (2007). 17486091 10.1038/ni1468

[b51] Sreejit G. . The ESAT-6 Protein of Mycobacterium tuberculosis Interacts with Beta-2-Microglobulin (β2M) Affecting Antigen Presentation Function of Macrophage. PLoS Pathog 10, e1004446 (2014). 25356553 10.1371/journal.ppat.1004446 PMC4214792

[b52] Welin A. . Culture filtrate protein 10 kDa (CFP-10) from Mycobacterium tuberculosis selectively activates human neutrophils through a pertussis toxin-sensitive chemotactic receptor. Infect Immun, doi: 10.1128/IAI.02493-14 (2014). PMC4288871 25332123

[b53] Cambier C. J. . Mycobacteria manipulate macrophage recruitment through coordinated use of membrane lipids. Nature 505, 218–222 (2014). 24336213 10.1038/nature12799 PMC3961847

[b54] Volkman H. E. . Tuberculous granuloma induction via interaction of a bacterial secreted protein with host epithelium. Science 327, 466–469 (2010). 20007864 10.1126/science.1179663 PMC3125975

[b55] Rahman A., Sobia P., Gupta N., Kaer L. V. & Das G. Mycobacterium tuberculosis subverts the TLR-2-MyD88 pathway to facilitate its translocation into the cytosol. PLoS ONE 9, e86886 (2014). 24475192 10.1371/journal.pone.0086886 PMC3903598

[b56] Watson R. O., Manzanillo P. S. & Cox J. S. Extracellular M. tuberculosis DNA Targets Bacteria for Autophagy by Activating the Host DNA-Sensing Pathway. Cell 150, 803–815 (2012). 22901810 10.1016/j.cell.2012.06.040 PMC3708656

[b57] Watson R. O. . The Cytosolic Sensor cGAS Detects Mycobacterium tuberculosis DNA to Induce Type I Interferons and Activate Autophagy. Cell Host Microbe 17, 811–819 (2015). 26048136 10.1016/j.chom.2015.05.004 PMC4466081

[b58] Wassermann R. . Mycobacterium tuberculosis Differentially Activates cGAS- and Inflammasome-Dependent Intracellular Immune Responses through ESX-1. Cell Host Microbe 17, 799–810 (2015). 26048138 10.1016/j.chom.2015.05.003

[b59] Collins A. C. . Cyclic GMP-AMP Synthase Is an Innate Immune DNA Sensor for Mycobacterium tuberculosis. Cell Host Microbe 17, 820–828 (2015). 26048137 10.1016/j.chom.2015.05.005 PMC4499468

[b60] Manzanillo P. S., Shiloh M. U., Portnoy D. A. & Cox J. S. Mycobacterium tuberculosis activates the DNA-dependent cytosolic surveillance pathway within macrophages. Cell Host Microbe 11, 469–480 (2012). 22607800 10.1016/j.chom.2012.03.007 PMC3662372

[b61] Cambier C. J., Falkow S. & Ramakrishnan L. Host evasion and exploitation schemes of Mycobacterium tuberculosis. Cell 159, 1497–1509 (2014). 25525872 10.1016/j.cell.2014.11.024

[b62] Majlessi L. & Brosch R. Mycobacterium tuberculosis Meets the Cytosol: The Role of cGAS in Anti-mycobacterial Immunity. Cell Host Microbe 17, 733–735 (2015). 26067600 10.1016/j.chom.2015.05.017

[b63] Andersen P. Tuberculosis vaccines - an update. Nature Reviews Microbiology 5, 484–487 (2007). 10.1038/nrmicro1703 17571458

[b64] Sun J., Vernier G., Wigelsworth D. J. & Collier R. J. Insertion of anthrax protective antigen into liposomal membranes: effects of a receptor. J Biol Chem 282, 1059–1065 (2007). 17107945 10.1074/jbc.M609869200

[b65] Sun J., Lang A. E., Aktories K. & Collier R. J. Phenylalanine-427 of anthrax protective antigen functions in both pore formation and protein translocation. Proc Natl Acad Sci USA 105, 4346–4351 (2008). 18334631 10.1073/pnas.0800701105 PMC2393744

[b66] Takaki K., Davis J. M., Winglee K. & Ramakrishnan L. nprot.2013.068. Nat Protoc 8, 1114–1124 (2013). 23680983 10.1038/nprot.2013.068 PMC3919459

