# Supplementary material for: EsxA membrane-permeabilizing activity plays a key role in mycobacterial cytosolic translocation and virulence: effects of single-residue mutations at glutamine 5
Source: Sci Rep. 2016 Sep 7;6:32618. doi: 10.1038/srep32618 (PMC5013644; doi:10.1038/srep32618)
Supplement: Supplementary Information [file srep32618-s1.pdf]

EsxA membrane-permeabilizing activity plays a key role in mycobacterial cytosolic translocation and virulence: effects of single mutation at glutamine 5

Qi Zhang[1,3], Decheng Wang[2,5], Guozhong Jiang[1,4], Wei Liu[1,4], Qing Deng[2], Xiujun Li[6], Wei Qian[7], Hugues Ouellet[1], Jianjun Sun[1,7]

Table S1. The p-values calculated by Log-Rank analysis of zebra fish embryos survival (* p <0.05).

	Mm	Mm ΔEsxA/B	Mm ΔEsxA/B(WT)	Mm ΔEsxA/B(Q5K)	Mm ΔEsxA/B(Q5V)
Mm	-	0.00018*	0.178	0.00114*	0.489
Mm ΔEsxA/B		-	0.013*	0.585	1.7e-05*
Mm ΔEsxA/B(WT)			-	0.0496*	0.0463*
Mm ΔEsxA/B(Q5K)				-	0.000127*
Mm ΔEsxA/B(Q5V)					-

Table S2: Sequences of the primers for *esxA*/*esxB* knockout in *Mm* and for PCR confirmation

Primers	Forward
KO-Left Arm-F (P_{LA}1)	5'-GAATTCCTGCAGGATGCCTTCAACGAGATGGATTAC-3'
KO-Left Arm-R (P_{LA}2)	5'-CTCTAGAGGATCCCCCGGCATCGGTCTTCATCTCT-3'
KO-Right Arm-F (P_{RA}1)	5'-CTCTAGAGGATCCCCGAACCTGGCTCGCACCATC–3'
KO-Right Arm-R (P_{RA}2)	5'-GACTAGAGGGTACCCTTCTTCTGCTATGTCCTGTTG -3'
PCR-Left Arm-F-1	5'-TAGAAGTCAGCGACGGCGCAGCCAG-3'
PCR-Left Arm-R-1	5'- CGTGGTCGAGGCGATGGCGGAG-3'
PCR-Left Arm-F-2	5'-TCGAACTGTACCGGATTTCG-3'

PCR-Right Arm-R-2	5'-GCTGATGGGCTTCGGTTG-3'
PCR-P_{CE1}	5'-CAGCATGGCAGAGATGAAGA-3'
PCR-P_{CE2}	5'-CGGTATTCTACGCGAACGAG-3'

Supplemental Figure 1

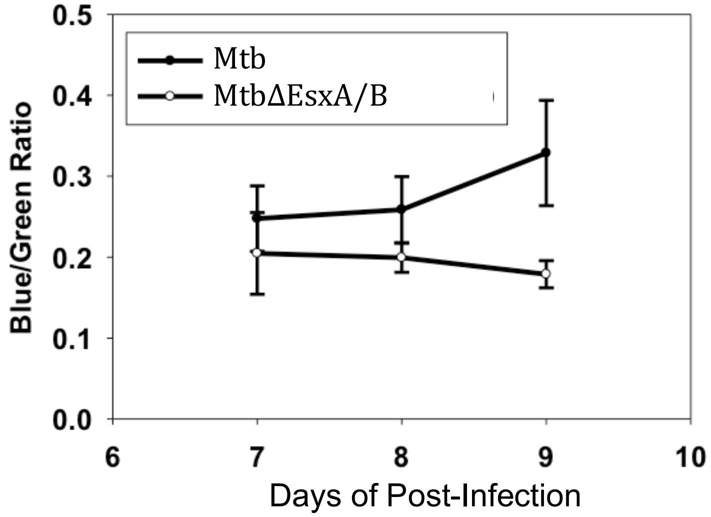

Figure S1. Time course of cytosolic translocation of Mtb and MtbΔEsxA/B in THP-1 cells measured by a fluorometer. THP-1 cells were infected with Mtb or MtbΔEsxA/B at MOI of 2. At the 7, 8 and 9 days of post-infection, the cells were loaded with CCF4-AM for 2 h. The cells were washed, detached and fixed by PFA. Blue (450nm)/green (530) ratio was measured in the fluorometer with excitation at 409 nm. The data is presented as means ± S.E.
